# Experimental Lung Ultrasound Scoring in a Murine Model of Aspiration Pneumonia: Challenges and Diagnostic Perspectives

**DOI:** 10.3390/diagnostics16020361

**Published:** 2026-01-22

**Authors:** Ching-Wei Chuang, Wen-Yi Lai, Kuo-Wei Chang, Chao-Yuan Chang, Shang-Ru Yeoh, Chun-Jen Huang

**Affiliations:** 1 Graduate Institute of Clinical Medicine, College of Medicine, Taipei Medical University, Taipei 110301, Taiwan; paindr.mali@gmail.com (C.-W.C.); 106207@w.tmu.edu.tw (W.-Y.L.); kenson7296@tmu.edu.tw (K.-W.C.); yuanc669@gmail.com (C.-Y.C.); 2 Department of Anesthesiology, School of Medicine, College of Medicine, Taipei Medical University, Taipei 110301, Taiwan; 3 Department of Anesthesiology, Wan Fang Hospital, Taipei Medical University, Taipei 116081, Taiwan; 4 Department of Integrative Research Center for Critical Care, Wan Fang Hospital, Taipei Medical University, Taipei 116081, Taiwan; 5 Center for Regional Anesthesia and Pain Medicine, Wan Fang Hospital, Taipei Medical University, Taipei 116081, Taiwan; 6 Department of Medical Research, Wan Fang Hospital, Taipei Medical University, Taipei 116081, Taiwan

**Keywords:** aspiration pneumonia, lung ultrasound, murine lung injury model, histological injury score, quantitative imaging, regional analysis, preclinical respiratory diagnostics, MLEUS

## Abstract

**Background:** Aspiration pneumonia (AP) remains a major cause of morbidity and mortality, yet non-invasive tools for monitoring lung injury in preclinical models are limited. Lung ultrasound (LUS) is widely used clinically, but existing murine scoring systems lack anatomical resolution and have not been validated for aspiration-related injury. **Methods:** We developed the Modified Lung Edema Ultrasound Score (MLEUS), a region-structured adaptation of the Mouse Lung Ultrasound Score (MoLUS), designed to accommodate the heterogeneous and gravity-dependent injury patterns characteristic of murine AP. Male C57BL/6 mice were assigned to sham, 6 h, 24 h, or 48 h groups. Regional LUS findings were compared with histological injury scores and wet-to-dry (W/D) ratios. Inter-rater reliability was assessed using the intraclass correlation coefficient (ICC). **Results:** Global LUS–histology correlation was weak (ρ = 0.33, *p* = 0.114). In contrast, regional performance varied markedly. The right upper (RU) zone showed the strongest correspondence with histological injury (r = 0.55, *p* = 0.005), whereas right and left diaphragmatic regions demonstrated minimal association. LUS abnormalities were detectable as early as 6 h, preceding clear histological progression. Inter-rater reliability was good (ICC = 0.87). **Conclusions:** MLEUS provides a reproducible, region-specific framework for evaluating aspiration-induced lung injury in mice. Although global correlations with histology were limited, region-dependent analysis identified that the RU zone as a reliable acoustic window for concurrent injury assessment. Early ultrasound changes highlight the sensitivity of LUS to dynamic aeration and interstitial alterations rather than cumulative tissue damage. These findings support the use of LUS as a complementary, non-invasive physiological monitoring tool in small-animal respiratory research and clarify its methodological scope relative to existing scoring frameworks.

## 1. Introduction

Aspiration pneumonia (AP) is a significant cause of morbidity and mortality, especially among critically ill patients with impaired consciousness or dysphagia [1,2,3,4]. The resulting pathophysiological cascade, including diffuse alveolar damage, pulmonary edema, and inflammatory infiltration, leads to highly variable and unpredictable clinical courses [1,5,6]. Early and accurate assessment of injury severity is therefore essential for guiding interventions and evaluating therapeutic strategies [7,8,9].

Murine models provide a tractable and physiologically relevant platform for studying aspiration-induced lung injury because they combine genetic manipulability, cost-effectiveness, and pulmonary architecture comparable to humans [10,11,12]. Both acid aspiration and infectious aspiration models demonstrate gravity-dependent and anatomically asymmetric injury, with dependent lobes such as the right inferior region being preferentially affected [13,14,15]. Similar positional effects have been reported in large-animal pneumonia models [16]. These observations highlight the need for region-specific evaluation tools in small-animal research. Aspiration injury is particularly sensitive to gravitational effects because instilled material settles in dependent lung regions, creating markedly asymmetric injury patterns that must be considered when designing and interpreting imaging-based assessments.

Histopathology remains the gold standard for assessing experimental lung injury, but its invasive nature prevents longitudinal monitoring [11]. Lung ultrasound (LUS) offers a rapid and non-invasive alternative and is widely used in the clinical evaluation of pulmonary edema, ARDS, and pneumonia [17,18,19]. However, most LUS scoring systems applied in murine studies are adapted directly from human or large-animal frameworks and lack the anatomical resolution needed for small-animal imaging [20,21,22,23,24,25,26]. Quantitative approaches such as quantitative lung ultrasound score (QLUSS) have improved scoring reproducibility [27], yet they remain limited to B-line quantification and do not account for heterogeneous inflammatory patterns.

The Mouse Lung Ultrasound Score (MoLUS) represented a methodological advance for evaluating cardiogenic pulmonary edema in mice [28]. Even so, MoLUS was developed for diffuse and symmetric edema, does not incorporate anatomical zoning or diaphragmatic windows, and has not been validated in aspiration pneumonia. Retrospective rescoring in our dataset further showed negligible correlation between MoLUS and histological injury, indicating limited applicability in this context.

To address these limitations, we developed the Modified Lung Edema Ultrasound Score (MLEUS), a region-structured adaptation of MoLUS tailored to the gravity-dependent and anatomically heterogeneous injury patterns characteristic of murine aspiration pneumonia [12,29]. MLEUS incorporates four anatomically anchored ventral zones and standardized diaphragmatic windows to improve spatial alignment between ultrasound findings, histology, and wet-to-dry ratios. To contextualize its methodological performance, all ultrasound recordings were additionally rescored using the original MoLUS protocol, allowing direct comparison between the two scoring frameworks.

While aspiration-induced lung injury is anatomically heterogeneous and strongly influenced by gravitational distribution, the physiological processes that precede overt tissue destruction, such as dynamic aeration loss and interstitial fluid shifts, are not necessarily mirrored by terminal histopathological severity. Lung ultrasound primarily interrogates these early physiological alterations rather than cumulative structural injury. Accordingly, the interpretation of region-specific ultrasound signals must consider not only injury distribution, but also signal stability and acoustic window reliability, particularly in small-animal models.

Using a validated murine model of aspiration pneumonia [29], we applied the MLEUS framework to evaluate the relationship between regional LUS findings and established markers of lung injury, including histological severity scores and wet-to-dry ratios. Ultrasound-defined scanning zones were anatomically matched to their corresponding histological sampling regions to ensure spatial consistency between imaging and ex vivo validation. Although LUS is widely used in clinical care, this study focuses on methodological rather than clinical questions. Our goal is to refine the technical application of lung ultrasound in a small-animal aspiration model and to strengthen its relevance for mechanistic studies of heterogeneous lung injury while enabling non-invasive longitudinal assessment.

## 2. Materials and Methods

### 2.1. Animal Ethics and Experimental Design

All animal procedures were approved by the Institutional Animal Care and Use Committee (IACUC) of Taipei Medical University (LAC2024-0418) and followed the 3R principles (Replacement, Reduction, and Refinement). Male C57BL/6 mice (8–10 weeks old, 20–25 g) were housed in pathogen-free conditions with free access to food and water under a 12 h light–dark cycle in temperature- and humidity-controlled, pathogen-free housing. Mice were randomly assigned into four groups (*n* = 6 per group): Sham (saline control), AP6 (sacrificed 6 h post-aspiration), AP24 (24 h), and AP48 (48 h).

### 2.2. Aspiration Pneumonia Model

The aspiration injury protocol employed in this study has been previously characterized and validated for both histopathological consistency and translational applicability in murine models (Chuang et al., 2025) [29]. This model replicates macro-aspiration-induced chemical and inflammatory lung injury commonly observed in clinical AP. Aspiration pneumonia was induced via oropharyngeal instillation of 50 μL of a synthetic gastric content administered under 3% isoflurane anesthesia. The instillate was composed of xanthan-based thickener, pepsin, and lipopolysaccharide (LPS), acidified to pH 1.6, simulating macro-aspiration-related injury. The Sham group received an equal volume of sterile saline.

### 2.3. Lung Ultrasound Scoring

Lung ultrasound (LUS) was performed to semi-quantitatively assess the severity of lung edema and injury in mice. A modified LUS scoring system, termed the Modified Lung Edema Ultrasound Score for Mice (MLEUS), was developed based on existing literature—particularly the Mouse Lung Ultrasound Score (MoLUS) introduced for evaluating pulmonary edema in murine heart failure models [28]. Targeted adaptations were made to address the characteristics of aspiration pneumonia and practical constraints in murine models. For each mouse, ultrasound clips were acquired and labeled by predefined zones to support region-specific scoring and downstream correlation with region-matched histology.

## 3. Ultrasound Acquisition

### 3.1. Equipment and Setup

Lung ultrasound (LUS) was performed using a high-frequency 40 MHz PB406e transducer (Prospect 3.0 with S-Sharp technology; S-Sharp Corporation, New Taipei City, Taiwan) connected to an ultra-high-resolution small-animal imaging system. Mice were positioned supine on a heated imaging platform, with body temperature maintained at 37 °C via an integrated temperature-control system. Anesthesia was maintained through a nose cone delivering 1.5–2% isoflurane in 100% oxygen, allowing spontaneous breathing while minimizing motion artifacts.

For diaphragmatic zones (RD and LD), the platform was tilted upward at a 10–20° angle at the tail end to optimize the acoustic window to the diaphragm and lung base. The probe was applied at the inferolateral subcostal angle and oriented obliquely, nearly perpendicular to the manubrium of the sternum. Ultrasound coupling gel was generously applied across the chest and subdiaphragmatic regions.

For the upper zones (RAU and LAU), scanning was performed more cranially along the anterolateral chest wall, approximately at or slightly lateral to the mid-clavicular line. This placement avoided interference from the cardiac silhouette while providing a stable acoustic window. The probe was oriented vertically, perpendicular to the chest surface, to visualize the pleural line and adjacent intercostal spaces.

All imaging conditions were standardized across animals and time points to ensure reproducibility.

### 3.2. Scanning Protocol and Zoning

To limit procedural stress, all imaging was completed within 10 min per mouse. Scans were performed in a supine position without repositioning, in accordance with ethical principles and 3R (Replacement, Reduction, Refinement) guidelines.

To capture aspiration-induced, gravity-dependent injury, four ventral zones were predefined according to anatomical orientation and diaphragmatic proximity:

• RAU: Right anteroventral upper
• RAL (RD): Right anteroventral lower/right diaphragmatic
• LAU: Left anteroventral upper
• LAL (LD): Left anteroventral lower/left diaphragmatic

**Figure 1:**
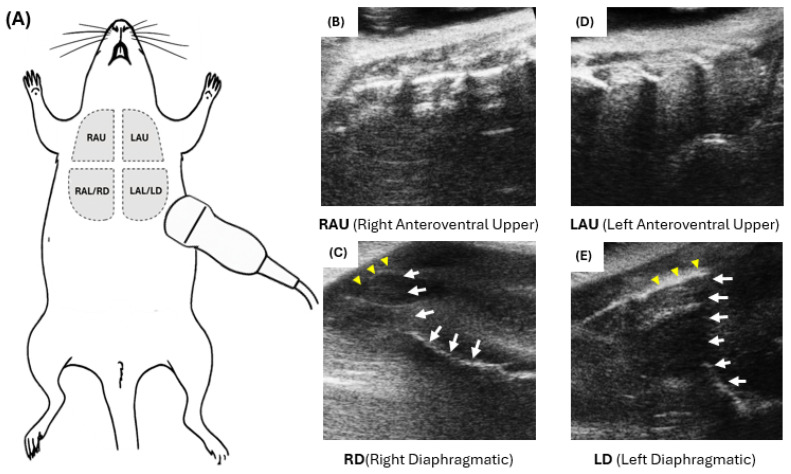
MLEUS ventral scanning zones and representative B-mode images. (**A**) Schematic showing the four ultrasound scanning zones on the ventral thorax of a supine mouse: RAU (right anteroventral upper), RAL/RD (right anteroventral lower/right diaphragmatic), LAU (left anteroventral upper), and LAL/LD (left anteroventral lower/left diaphragmatic). (**B**–**E**) Representative B-mode images from each zone. Yellow arrowheads indicate costal pleura; white solid arrows denote the lung base and diaphragmatic pleura, forming the pleuro-diaphragmatic angle. Images illustrate zone-specific acoustic windows used for scoring, not specific injury grades.

Figure 1 shows schematic and representative B-mode images illustrating these scanning zones. These zones were mapped to their corresponding anatomical lung lobes (see Supplementary Table S1), providing anatomical consistency across LUS, histology, and gravimetric analysis.

#### 3.2.1. Zone Acquisition and Recording Handling

Each mouse was scanned sequentially across all four predefined zones, and cine loops were saved and labeled by zone at the time of acquisition. Because of the small thoracic dimensions and limited acoustic windows in mice, occasional recordings captured overlapping fields (most commonly LU and adjacent diaphragmatic views). To ensure methodological consistency, we applied the following a priori handling rules:

(i) LU-only views were not used for correlation analyses due to poor reproducibility in pilot assessments;
(ii) Mixed LU + LD recordings were retained only for the diaphragmatic (LD) portion, because the pleuro-diaphragmatic angle and lung base remained consistently identifiable, whereas the LU component was more susceptible to rib shadowing and cardiac-motion artifacts; thus, these mixed recordings were reassigned to LD for analysis;
(iii) Regions demonstrating poor reproducibility were excluded from quantitative correlation analyses to minimize interpretive noise.

#### 3.2.2. Rationale for LU Exclusion

Pilot reproducibility assessments across three blinded operators suggested low agreement for LU (qualitative ICC estimates < 0.40), primarily due to inconsistent rib shadows, interference from cardiac motion, and narrower acoustic windows compared with other ventral zones. Therefore, final regional correlation analyses were restricted to RU, RD, and LD.

### 3.3. Regional Lung Ultrasound (LUS) Analysis

LUS examinations were performed in anesthetized mice using a small-animal high-frequency PB406e probe on the Prospect 3.0 platform. Each zone was scored on a 0–3 scale based on aeration loss and pleural/subpleural abnormalities (see Section 4). For quantitative analyses, regional correlations with histology were performed using the reproducible ventral zones (RU, RD, and LD) according to the predefined acquisition and data-handling rules described above.

## 4. MLEUS Scoring System

### 4.1. Criteria and Interpretation

**Figure 2:**
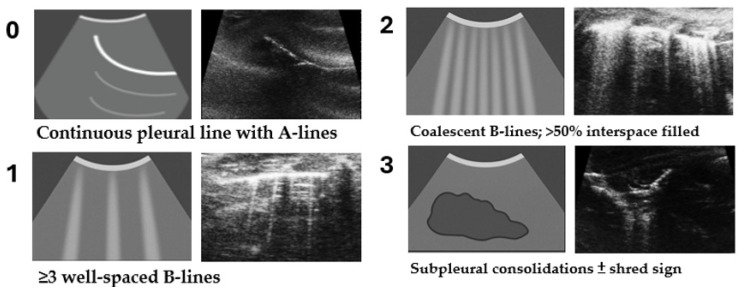
Schematic and representative ultrasound images illustrating the MLEUS scoring criteria (0–3). Score 0: continuous pleural line with horizontal A-lines and no B-lines. Score 1: ≥3 well-spaced vertical B-lines. Score 2: coalescent B-lines occupying >50% of the intercostal space. Score 3: subpleural consolidation with or without shred sign. Left panels show schematic diagrams of key features; right panels show corresponding B-mode images acquired with a 40 MHz probe under standardized settings.

A Modified Lung Edema Ultrasound Score (MLEUS) was developed based on pleural–subpleural artifacts and aeration patterns. Each zone was graded on a 0–3 scale (Figure 2).

Each mouse received a zone-specific score (0–3), yielding a maximum composite score of 12 (see Table 1). Although the LU zone was excluded during statistical correlation analyses, all four zones were still scored during acquisition to maintain consistency with the original four-zone scoring structure. To emphasize parenchymal aeration and edema, the scoring excluded pleural effusion, pleural thickening, and Z-lines, minimizing confounding factors. Representative ultrasound images for each score are summarized in Figure 2.

### 4.2. Inter-Rater Reliability and Blinding

Two blinded investigators independently reviewed and scored all LUS images and video clips. Both were blinded to group assignment and timepoint. Discrepancies > 1 point were resolved by consensus or arbitration by a third reviewer. Inter-rater reliability, assessed using the intraclass correlation coefficient (ICC) [30], demonstrated good agreement (ICC = 0.87, 95% CI 0.76–0.94), confirming the reproducibility of the MLEUS system.

### 4.3. Validation of MLEUS Score

To validate the MLEUS scoring system, ultrasonographic findings were compared with two established markers of pulmonary injury: the lung wet-to-dry (W/D) weight ratio and histological injury scores. Lung tissue samples were collected from the same ventral regions assessed by LUS to ensure spatial consistency. Histological sampling was regionally matched to the corresponding ultrasound zones (see Supplementary Table S1), enabling direct correlation analyses between imaging, gravimetric, and histopathological markers.

### 4.4. Wet-to-Dry Weight Ratio (W/D Ratio)

At designated timepoints, mice were euthanized via isoflurane overdose followed by exsanguination. Lungs were excised en bloc, gently blotted, and weighed (wet weight). Samples were dried at 60 °C for 48 h to obtain dry weight. The W/D ratio was calculated and used as a quantitative index of pulmonary edema. Because the wet to dry assay is terminal and requires removal of the entire lung, sampling was limited to three lungs per time point in accordance with IACUC and reduction principles. Wet to dry measurements cannot be assigned to specific regions within a single mouse, so correlations were evaluated at the time point mean level rather than on a per mouse basis.

### 4.5. Histological Lung Injury Score

Formalin-fixed, paraffin-embedded lung sections from corresponding LUS regions were stained with hematoxylin and eosin (H&E) and scored using a weighted system adapted from Matute-Bello et al. (2001) [11]:

A: Neutrophils in alveolar space (0–2)

B: Neutrophils in interstitial space (0–2)

C: Hyaline membranes (0–2)

D: Proteinaceous debris (0–2)

E: Septal thickening (0–2)

Total score = [(20 × A) + (14 × B) + (7 × C) + (7 × D) + (2 × E)] ÷ (number of fields × 100)

All slides were analyzed in a blinded fashion by two independent operators.

The established Matute-Bello weighting scheme was adopted without modification to preserve comparability with prior ALI/ARDS preclinical studies, ensuring that our results can be contextualized within existing literature.

### 4.6. Definition of Injury Severity

Mice were stratified into two outcome groups to facilitate ROC analysis: mild injury and moderate-to-severe injury, based on their histological lung injury scores. Without established histopathological severity benchmarks for aspiration pneumonia in mice, we adopted a data-driven threshold defined as the median histological score across all animals (0.3167). Mice with scores ≥ 0.3167 were categorized as having moderate-to-severe injury. This approach aligns with standard practices in preclinical diagnostic studies, where median-based stratification is frequently applied when no external reference cutoffs are available.

This approach is commonly applied in preclinical diagnostic studies, where median-based stratification or ROC-derived thresholds are used to dichotomize continuous variables without established cutoffs. It provides internal consistency and balanced group sizes, facilitating exploratory ROC analyses in small-sample experimental designs [31].

### 4.7. Statistical Analysis

The primary endpoint of this study was the correlation between regional LUS scores and histological lung injury severity. Secondary endpoints included global LUS correlations, agreement analyses between scoring systems, and exploratory ROC performance. All statistical analyses were conducted using GraphPad Prism 10.0 and SPSS 28.0. Data were expressed as median ± interquartile range (IQR) unless otherwise specified.

• Group comparisons were performed using the Kruskal–Wallis test followed by Dunn’s multiple comparisons test.
• Pairwise comparisons were conducted using the Mann–Whitney U test.
• Spearman’s rank correlation was applied to evaluate associations between MLEUS scores, W/D ratios, and histological scores.
• Receiver Operating Characteristic (ROC) analysis was used in an exploratory fashion to assess the diagnostic performance of regional LUS scores. ROC analysis was performed on the composite LUS score rather than on individual regions because the sample size was insufficient for reliable zone-specific ROC curves. The ROC evaluation was exploratory and intended only to provide a global assessment of LUS discrimination. Area under the curve (AUC) values were interpreted as follows: 0.7–0.8 (acceptable), 0.8–0.9 (excellent), and >0.9 (outstanding). Statistical significance was defined as *p* < 0.05.

### 4.8. Minimum Criteria Rationale for Scoring System Design

The Modified Lung Edema Ultrasound Score (MLEUS) was deliberately simplified to focus on ultrasonographic features most consistently associated with pulmonary edema and aeration loss. Building on prior frameworks such as the MoLUS, we intentionally excluded pleural effusion, pleural thickening, and Z-lines. These features are highly operator-dependent in murine models, prone to variability from anesthesia and positioning, and lack consistent histopathological validation, thereby introducing confounding noise. Instead, the MLEUS emphasized reproducible parenchymal aeration markers—namely B-lines and subpleural consolidation—that demonstrated closer alignment with histological injury and wet-to-dry (W/D) ratios.

▪ The final system was designed to satisfy three essential methodological criteria:
▪ Clearly defined, region-based scoring metrics to ensure anatomical consistency.
▪ Inter-rater reliability formally assessed and reported to confirm reproducibility.
▪ Measurable correlations with histopathological and physiological indices of lung injury, providing construct validity.

By narrowing its scope to features with the strongest biological relevance and reproducibility, MLEUS improved correlation fidelity, reduced interpretative ambiguity, and aligned with clinical LUS practices that prioritize rapid and reliable assessment of parenchymal changes over ancillary pleural signs. Pleural effusion, pleural thickening, and Z-lines were not included because these findings are highly operator dependent in small-animal imaging and lack validated histopathological correspondence in murine aspiration models. Including these features would increase interpretive variability without improving diagnostic clarity.

### 4.9. Use of Generative Artificial Intelligence (GenAI)

Generative artificial intelligence (GenAI) tools, including ChatGPT-4 (OpenAI, San Francisco, CA, USA), were used to assist in text drafting, language editing, and figure layout optimization. All outputs were critically reviewed, revised, and validated by the authors to ensure accuracy and scientific integrity. GenAI was not used for study design, data collection, or primary statistical analysis.

## 5. Results

### 5.1. Overview of Study Findings

Using the Modified Lung Edema Ultrasound Score (MLEUS) framework, we systematically evaluated aspiration-induced lung injury in mice through multi-modal analyses. To validate construct validity, regional lung ultrasound (LUS) scores were assessed and compared with histological injury scores and wet-to-dry (W/D) ratios. The results are presented in sequential order, beginning with overall correlations across modalities, followed by temporal progression of lung injury, regional heterogeneity in LUS–histology correspondence, diagnostic performance based on receiver operating characteristic (ROC) analysis, and histopathological validation. This structured presentation highlights the complementary strengths and limitations of LUS relative to established injury markers.

#### 5.1.1. Early Detection of Pulmonary Injury

**Figure 3:**
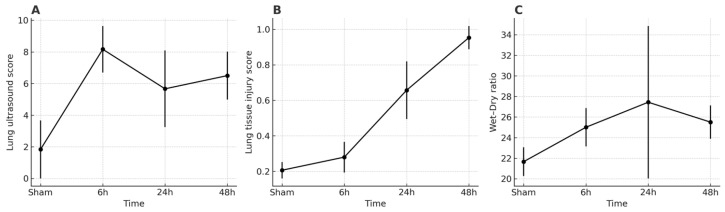
Temporal trends of lung injury severity across three modalities. Lung injury severity was assessed in a murine model of aspiration pneumonia using three complementary modalities across four time points (Sham, 6 h, 24 h, 48 h). (**A**) Lung ultrasound score (LUS), reflecting aeration loss and pleural abnormalities, increased significantly at 6 h and remained elevated thereafter (*n* = 6 per group). (**B**) Histology-based lung tissue injury score, representing alveolar damage and inflammatory cell infiltration, showed a progressive increase from Sham to 48 h (*n* = 6 per group). (**C**) Wet–Dry ratio, an index of pulmonary edema, peaked at 24 h with greater inter-individual variability and remained elevated at 48 h (*n* = 3 per group). Data are presented as mean ± SD. Error bars indicate standard deviation.

Regional lung ultrasound (LUS) scores detected abnormalities as early as 6 h post-aspiration, indicating sensitivity to early physiological changes (Figure 3A). The interpretation of these early alterations is further addressed in the Discussion.

#### 5.1.2. Temporal Progression and Regional Susceptibility

**Figure 4:**
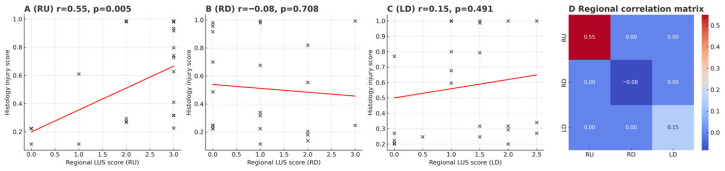
Regional correlations between lung ultrasound (LUS) and histology injury scores. (**A**) Scatter plot of regional LUS versus histology scores in the right upper (RU) lung region (*n* = 24), showing a significant positive correlation (*r* = 0.55, *p* = 0.005). (**B**) Right diaphragmatic (RD) region (*n* = 23), showing no significant correlation (*r* = –0.08, *p* = 0.71). (**C**) Left diaphragmatic (LD) region, including combined LU+LD zones (*n* = 24), showing a weak non-significant correlation (*r* = 0.15, *p* = 0.49). (**D**) Heatmap summarizing correlation coefficients across regions.

From 6 to 48 h, LUS trajectories were heterogeneous across the predefined ventral zones (Figure 3A). In regional analyses, the right upper (RU) zone consistently showed the strongest concordance with histological injury (r = 0.55, *p* = 0.005, *n* = 24), underscoring RU as the most reliable acoustic window (Figure 4A,D). By contrast, the right diaphragmatic (RD) zone displayed LUS elevations without corresponding histological severity (r = −0.08, *p* = 0.71; Figure 4B), and the left diaphragmatic (LD, including LU+LD) zone showed only weak, non-significant association (r = 0.15, *p* = 0.49; Figure 4C). Notably, while group-level LUS increased with injury progression (Figure 3A), the wet-to-dry (W/D) ratio peaked at 24 h with larger inter-individual variability (Figure 3C), consistent with dynamic edema redistribution. These findings highlight that regional heterogeneity and temporal dynamics critically shape LUS interpretability, with RU emerging as the most dependable region for concurrent injury readout.

#### 5.1.3. Correlation with Established Injury Markers

**Figure 5:**
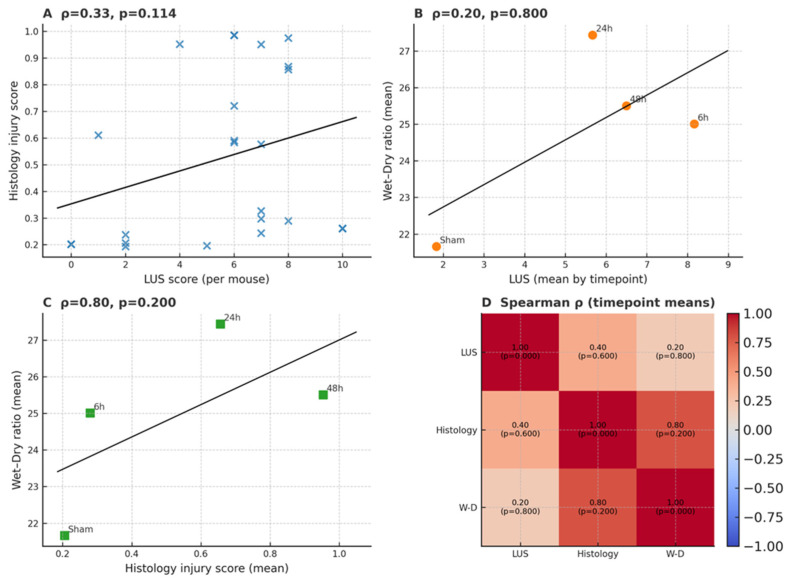
Correlation of lung ultrasound (LUS) with histological injury and wet-to-dry (W/D) ratios. (**A**) Scatterplot showing the correlation between per-mouse LUS scores and histological injury (*n* = 24), demonstrating a positive but non-significant association (ρ = 0.33, *p* = 0.114). (**B**) Correlation between timepoint means of LUS scores and W/D ratios (*n* = 4), showing a weak association (ρ = 0.20, *p* = 0.80). (**C**) Correlation between timepoint means of histological injury scores and W/D ratios (*n* = 4), showing a stronger trend (ρ = 0.80, *p* = 0.20). (**D**) Heatmap summarizing pairwise Spearman’s ρ values and *p* values across modalities, highlighting directional concordance despite limited statistical power.

Across all animals, per-mouse LUS scores showed a positive but non-significant association with histological injury (Spearman ρ = 0.33, *p* = 0.114; Figure 5A). Because wet-to-dry (W/D) ratios were measured at the whole-lung level (*n* = 3 per timepoint), correlations were assessed using timepoint means (*n* = 4). At this aggregate level, LUS(mean) vs. W/D(mean) demonstrated only a weak association (ρ = 0.20, *p* = 0.80; Figure 5B), whereas Histology(mean) vs. W/D(mean) exhibited a stronger trend (ρ = 0.80, *p* = 0.20; Figure 5C). A heatmap summary highlights these relationships across modalities (Figure 5D). Given the small sample size and limited degrees of freedom, these W/D-based comparisons should be regarded as exploratory. Nonetheless, the directional concordance supports the construct validity of MLEUS, indicating that LUS primarily captures dynamic aeration and interstitial alterations rather than cumulative tissue-level damage. Exact mean and standard deviation values for all ultrasound scores, histological injury scores, and wet to dry ratios are provided in Supplementary Table S4.

#### 5.1.4. Diagnostic Performance and Threshold-Based Prediction

**Figure 6:**
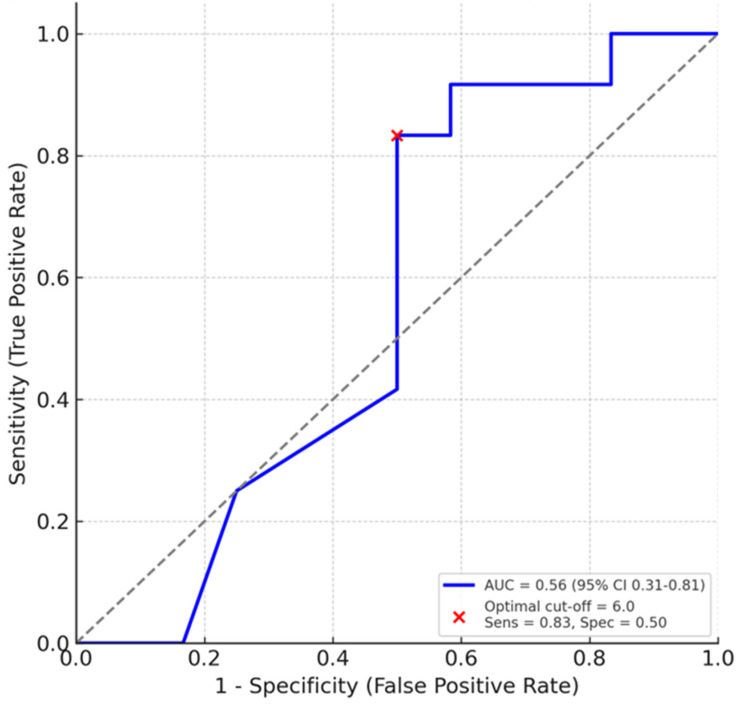
Diagnostic performance of LUS scoring. Receiver operating characteristic (ROC) curve for LUS in discriminating histology-defined moderate-to-severe lung injury (injury score ≥ 0.5). The area under the curve (AUC) was 0.56 (95% CI 0.31–0.81). The optimal cut-off was 6, yielding a sensitivity of 83% and a specificity of 50%.

Receiver operating characteristic (ROC) analysis was performed using a histology-defined dichotomy and per-mouse composite LUS (Figure 6). ROC analysis was limited to the global LUS score. Regional ROC curves were not generated because the sample size within each zone was insufficient to support meaningful threshold-based evaluation. The overall AUC was 0.56 (95% CI 0.31–0.81). An LUS cut-off of 6 yielded a sensitivity of 0.83 and a specificity of 0.50, indicating that while LUS is sensitive for detecting injured lungs, its discriminative specificity for excluding mild injury is limited in this dataset.

#### 5.1.5. Representative Histopathology

**Figure 7:**
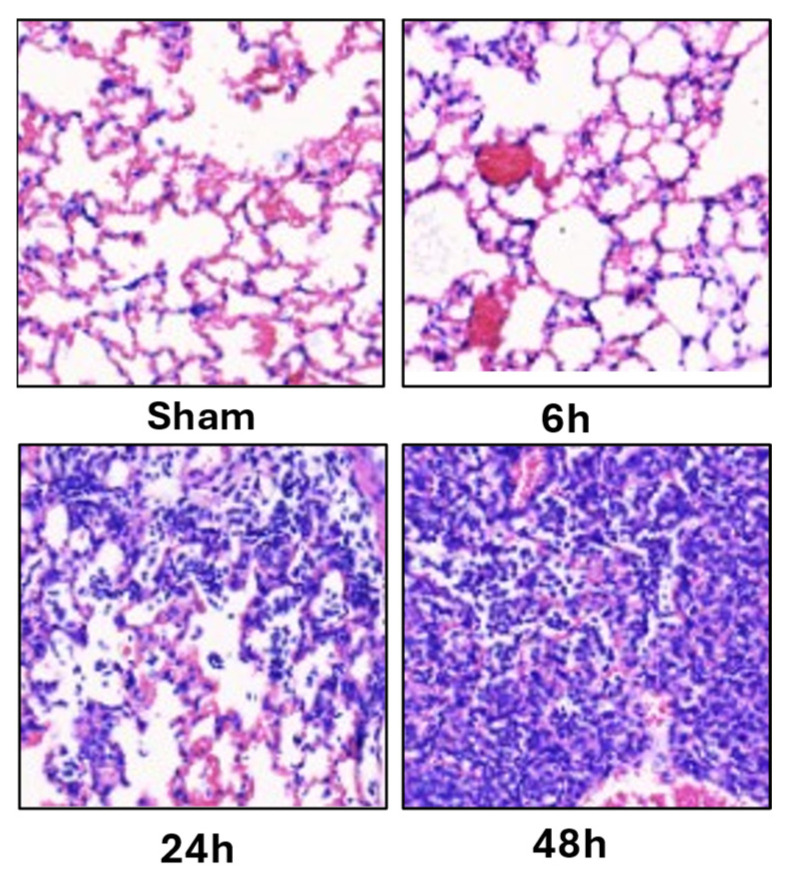
Representative histopathological changes across time points. Representative hematoxylin and eosin (H&E)-stained lung sections from Sham, 6 h, 24 h, and 48 h groups. Sham lungs displayed preserved alveolar architecture without inflammatory infiltrates. At 6 h, early interstitial edema and mild inflammatory cell accumulation were observed. At 24 h, alveolar wall thickening, hemorrhage, and neutrophil infiltration became apparent. At 48 h, severe alveolar disruption and diffuse inflammatory injury were evident. These morphological findings correspond to the progressive histology injury scores shown in Figure 3B.

Representative H&E sections illustrate the temporal evolution from preserved alveolar architecture in Sham to interstitial edema at 6 h, prominent wall thickening/hemorrhage/neutrophilic infiltration at 24 h, and diffuse architectural disruption at 48 h (Figure 7), visually validating the quantitative histology trajectory (Figure 3B) and the inter-modal trends in Figure 5.

For additional supporting data, extended statistical analyses, and supplementary figures, please refer to the Supplementary Materials.

## 6. Discussion

### 6.1. Region-Specific Reliability of LUS

In this murine model of aspiration pneumonia, the MLEUS framework demonstrated that the diagnostic performance of lung ultrasound (LUS) is inherently region dependent. While the global per-mouse LUS score showed only a weak association with histological severity, regional analysis revealed a clearer pattern: the right upper (RU) zone showed the strongest correspondence with tissue injury, whereas the right diaphragmatic (RD) and left diaphragmatic (LD) regions demonstrated little or no meaningful association.

Importantly, these findings indicate that regional ultrasound interpretability is shaped by both biological heterogeneity and acoustic window reliability. In small animals, signal stability varies across zones because rib shadowing, cardiac motion, and diaphragmatic excursion can differentially obscure pleural and subpleural patterns. Therefore, region-structured scoring is essential not only to reflect gravity-dependent injury heterogeneity, but also to identify regions that provide reproducible physiological signals suitable for longitudinal monitoring.

### 6.2. Diagnostic Correlation with Established Injury Markers

In line with prior reports on the application of LUS in pulmonary edema and acute lung injury (ALI) [17,32], we adapted the Modified Lung Edema Ultrasound Score (MLEUS) from the previously described Mouse Lung Ultrasound Score (MoLUS) protocols [33,34]. These refinements were specifically designed to capture the pathophysiological characteristics of murine aspiration pneumonia and the practical constraints of small-animal imaging. The resulting region-structured framework aimed to improve anatomical consistency and reduce interpretive noise from operator-dependent pleural artifacts, thereby supporting more reproducible quantification of aeration loss.

At the global level, LUS and histological injury scores showed only a weak, non-significant association. However, regional analysis demonstrated clearer patterns, with the RU zone consistently emerging as the most reliable acoustic window and showing the strongest correspondence with histological injury. This observation raises an important methodological consideration: the region with the greatest expected gravity-dependent injury burden is not necessarily the region that yields the most stable or interpretable ultrasound signal in mice.

In murine models, diaphragmatic zones can be disproportionately affected by technical limitations, including rib shadowing, diaphragmatic motion, and limited subcostal acoustic windows, which may reduce scoring reproducibility even when underlying pathology is substantial. In contrast, the RU region provides a comparatively stable acoustic window with reduced interference, allowing more consistent visualization of pleural-line integrity, B-lines, and subpleural consolidation.

Moreover, ultrasound primarily captures dynamic physiological changes in aeration and interstitial fluid rather than cumulative tissue-level injury measured by terminal histopathology. Therefore, partial discordance between regional LUS patterns and the distribution of histological injury should be expected in a heterogeneous, gravity-influenced aspiration model. Taken together, these findings support interpreting MLEUS as a complementary, physiology-sensitive tool whose performance is region dependent, with RU providing the most consistent concurrent injury readout under the present acquisition conditions.

From an early-monitoring perspective, this region-dependent reliability does not imply that injury occurs preferentially in the RU zone, nor that diaphragmatic regions are biologically less relevant. Rather, it delineates the methodological scope of murine LUS: early ultrasound signals may be detected most consistently in zones with stable acoustic windows, whereas the regions of maximal cumulative pathology may require alternative imaging strategies, refined windows, or complementary ex vivo validation.

### 6.3. Median-Based Thresholds as a Pragmatic Diagnostic Tool

To enable exploratory ROC analysis, mice were stratified into mild versus moderate-to-severe injury groups using the median histological score as an internal cutoff. This pragmatic approach is widely employed in preclinical diagnostic studies when external reference thresholds are lacking, as it ensures balanced group sizes and internal consistency. Using this cutoff, LUS achieved high sensitivity but limited specificity, consistent with its ability to detect injury presence rather than discriminate against its severity. These results support the use of LUS as an early, physiological monitoring biomarker, while emphasizing the need for future studies to validate more robust thresholds—such as those derived from Youden’s index, likelihood ratios, or Bayesian modeling—in larger cohorts and across additional aspiration paradigms or positioning strategies.

### 6.4. Temporal Sensitivity and Early Detection

LUS abnormalities were observed as early as 6 h post-aspiration, preceding overt histological progression in this model. This temporal dissociation suggests that ultrasound primarily captures early physiological changes in aeration and interstitial fluid that may occur before cumulative structural pathology becomes pronounced on terminal tissue scoring. Comparable findings have been reported in clinical and experimental settings, where ultrasound identifies interstitial syndrome or focal aeration loss earlier than other structural reference measures.

These findings support the use of murine LUS as an early physiological monitoring tool for evaluating dynamic injury evolution and therapeutic response. At the same time, early ultrasound signals should not be overinterpreted as direct surrogates of cumulative histological severity, particularly in heterogeneous aspiration models where edema redistribution and regional motion artifacts can influence signal appearance.

### 6.5. Reproducibility and Translational Implications

A notable strength of the MLEUS framework lies in its reproducibility. Inter-rater reliability was good (ICC = 0.87, 95% CI 0.76–0.94), confirming consistency across blinded investigators. By deliberately excluding pleural effusion, pleural thickening, and Z-lines, features prone to operator dependence and poorly validated in murine aspiration models, the scoring system was streamlined to emphasize robust parenchymal aeration markers, namely B-lines and subpleural consolidation. These refinements improved internal consistency and reduced interpretive ambiguity. Direct benchmarking against the original MoLUS scoring system further showed that, although both systems detect global aeration changes, MLEUS better reflects the heterogeneous injury patterns characteristic of aspiration pneumonia.

Importantly, a reproducible and region-structured LUS framework enables repeated, non-terminal assessments within the same animal, thereby supporting longitudinal study designs. This methodological advantage has direct ethical and translational implications: it may increase information yield per animal while reducing reliance on serial sacrifice, in accordance with the principles of Replacement, Reduction, and Refinement (3R). By facilitating early and dynamic physiological monitoring, MLEUS provides a practical platform for accelerating mechanistic and therapeutic studies in aspiration-related lung injury.

### 6.6. Limitations and Future Directions

While the present study provides methodological insights into the application of lung ultrasound in murine aspiration pneumonia, several limitations should be acknowledged. First, the use of mice introduces inherent technical constraints, including small thoracic dimensions and narrow acoustic windows that can limit the resolution and stability of lung ultrasound imaging. These challenges are well recognized in the literature and reflect well-recognized constraints of small-animal lung ultrasound rather than limitations specific to the present study. Second, LUS scoring remains semi-quantitative. Although MLEUS improves regional consistency, it cannot fully capture the complexity of histopathological injury, and partial discordance between imaging and tissue findings is expected. Third, aspiration pneumonia produces gravity-dependent and regionally heterogeneous injury. Even with standardized positioning and controlled instillation, biological variability cannot be eliminated and likely contributes to zone-to-zone differences in LUS performance. These constraints underscore the importance of explicitly defining the methodological scope of murine LUS rather than overextending its interpretative claims.

The aspiration pneumonia model itself also carries practical limitations. Experimental AP models suitable for repeated imaging or mechanistic evaluation remain relatively limited, and large-animal models, although offering more generous acoustic windows, are often constrained by ethical, financial, and logistical considerations. Moreover, standardizing aspiration injury in larger species poses additional challenges related to injury distribution and reproducibility. Consequently, murine models remain the most feasible and widely adopted platform for controlled studies of aspiration-related lung injury, provided that their technical and physiological boundaries are clearly acknowledged. Finally, the sample size for wet-to-dry measurements was limited to three lungs per time point because the assay is terminal and destructive. These data therefore served primarily as group-level physiological indicators rather than mouse-specific correlates. ROC analysis was exploratory and based on a global LUS score, and the modest cohort size limits generalizability. Larger studies across additional models of aspiration injury will be valuable for refining diagnostic thresholds and further validating the MLEUS framework.

Accordingly, the present findings should be interpreted as exploratory and hypothesis-generating with respect to diagnostic thresholds, rather than as definitive performance benchmarks.

## 7. Conclusions

The Modified Lung Edema Ultrasound Score (MLEUS) provides a structured and reproducible framework for assessing aspiration-induced lung injury in mice. Consistent with the gravity-dependent and regionally heterogeneous nature of aspiration pneumonia, global correlations between LUS and histological injury were limited. However, region-specific analysis revealed meaningful differences in ultrasound interpretability across acoustic windows. The right upper zone demonstrated the most consistent correspondence with histological injury, whereas diaphragmatic regions showed weaker or absent associations.

Importantly, LUS abnormalities were detectable as early as 6 h after aspiration, indicating sensitivity to early physiological alterations in lung aeration even when cumulative histological disruption remained modest. These findings reinforce the role of LUS as a complementary, physiologically responsive monitoring modality rather than a direct surrogate for terminal tissue injury. Good inter-rater reliability further supports the methodological robustness of the MLEUS framework.

Future studies incorporating larger cohorts, optimized acquisition strategies, and externally validated diagnostic thresholds will be essential for refining regional scoring approaches and expanding the applicability of LUS in preclinical pulmonary research. Continued development of anatomically grounded and physiology-aware scoring systems such as MLEUS may help advance longitudinal, non-invasive assessment strategies and support ethically responsible investigation of heterogeneous lung injury.

## Figures and Tables

**Table 1:** Semi-quantitative scoring criteria for the Modified Lung Edema Ultrasound Score (MLEUS). Scores from 0 to 3 were assigned to each predefined lung zone according to pleural-line appearance, B-line distribution, and subpleural consolidation, corresponding to increasing degrees of lung aeration impairment in mice.

Score	Ultrasound Features	Interpretation
0	Continuous pleural line with A-lines; no B-lines	Normal aeration
1	≥3 well-spaced B-lines	Mild interstitial syndrome
2	Coalescent B-lines; >50% interspace filled	Moderate alveolar-interstitial syndrome
3	Subpleural consolidations ± shred sign	Severe loss of aeration or alveolar flooding

## Data Availability

The datasets generated and analyzed during the current study (including LUS scores and summary histological indices) are available from the corresponding author on reasonable request. Raw ultrasound and histological image data are not publicly available due to ethical considerations.

## Supplementary Materials

The following supporting information can be downloaded at https://www.mdpi.com/article/10.3390/diagnostics16020361/s1, Table S1: Correspondence between Ultrasound Scanning Zones and Anatomical Lung Lobes in Mice; Table S2: Regional correlations between lung ultrasound (LUS) and histology injury scores; Table S3: MLEUS vs. MoLUS comparison table; Table S4: Descriptive statistics for all markers; Table S5: Statistical tests summary; Figure S1: Paired comparison MLEUS vs. MoLUS; Figure S2: Bland–Altman plot; Figure S3: Correlation heatmap.

## Abbreviations

The following abbreviations are used in this manuscript:

ALI	Acute Lung Injury
AP	Aspiration Pneumonia
AUC	Area Under the Curve
CI	Confidence Interval
H&E	Hematoxylin and Eosin
ICC	Intraclass Correlation Coefficient
IQR	Interquartile Range
LUS	Lung Ultrasound
MLEUS	Modified Lung Edema Ultrasound Score
MoLUS	Mouse Lung Ultrasound Score
RAU	Right Anteroventral Upper
RD (RAL)	Right Diaphragmatic (Right Anteroventral Lower)
ROC	Receiver Operating Characteristic
RU	Right Upper (zone)
LD (LAL)	Left Diaphragmatic (Left Anteroventral Lower, including LU+LD)
SD	Standard Deviation
SEM	Standard Error of the Mean
W/D ratio	Wet-to-Dry Weight Ratio
