# Supplementary material for: Experimental Lung Ultrasound Scoring in a Murine Model of Aspiration Pneumonia: Challenges and Diagnostic Perspectives
Source: Diagnostics (Basel). 2026 Jan 22;16(2):361. doi: 10.3390/diagnostics16020361 (PMC12839569; doi:10.3390/diagnostics16020361)
Supplement: Supplementary file 1 [file diagnostics-16-00361-s001.zip › diagnostics-3877092-supplementary.pdf]

Supplementary Table S1. Correspondence between Ultrasound Scanning Zones and Anatomical Lung Lobes in Mice

Ultrasound Zone (MLEUS)	Abbreviation	Approximate Anatomical Lobe(s) Represented	Notes / Limitations
Right Anteroventral Upper	RAU	Right Upper (RU) Lobe	Good acoustic window; correlates moderately with histology
Right Anteroventral Lower / Right Diaphragmatic	RAL / RD	Primarily Right Inferior (RI) Lobe	Overlap with diaphragm; poor correlation with histology in this study
Left Anteroventral Upper	LAU	Left Upper (LU) Lobe	Cardiac interference may reduce interpretability
Left Anteroventral Lower / Left Diaphragmatic	LAL / LD	Left Lower (LL) Lobe	Dependent region; weak correlation with histology

Supplementary Table S2. Regional correlations between lung ultrasound (LUS) and histology injury scores

Region	n	r (correlation coefficient)	p-value	Interpretation
RU	24	0.55	0.005	Moderate positive correlation, statistically significant
RD	23	–0.08	0.71	No correlation, not significant
LD (LU+LD)	24	0.15	0.49	Weak correlation, not significant

Table S2: Regional correlations between lung ultrasound (LUS) scores and histology injury scores. Pearson's correlation coefficients (r), p-values, and sample sizes (n) are shown for each lung region. RU = right upper, RD = right diaphragmatic, LD = left diaphragmatic (including combined LU+LD zones).

Supplementary Table S3: Direct comparison between MLEUS and MoLUS performance.

Comparison	Pearson r	Spearman ρ	p-value	n
**MoLUS vs Histology**	**0.03**	**0.05**	**0.81**	**24**
**MLEUS vs Histology**	**0.29**	**0.33**	**0.11**	**24**
**MLEUS vs MoLUS**	**0.72**	**0.69**	**<0.001**	**24**

Table S3. ***Comparison of MLEUS and MoLUS correlations with histology and wet-to-dry (W/D) ratios.*** This table presents Pearson and Spearman correlation coefficients between MLEUS, MoLUS, histology, and W/D ratios. MLEUS demonstrated directionally consistent and stronger correlations with histological severity compared with MoLUS, which showed negligible association (r ≈ 0.03). MLEUS and MoLUS were moderately correlated with each other (r ≈ 0.72), suggesting shared sensitivity to aeration loss, but only MLEUS aligned with biologically relevant injury patterns in aspiration pneumonia.

Supplementary Table S4: Descriptive statistics for all markers

Group	n	**MLEUS** (mean ± SD)	**MoLUS** (mean ± SD)	**Histology** (mean ± SD)	**W/D** (mean ± SD)
**Sham**	6	1.83 ± 1.83	17.17 ± 9.37	0.191 ± 0.004	21.66 ± 1.39
**AP(6 h)**	6	8.17 ± 1.47	29.33 ± 8.48	0.218 ± 0.012	25.01 ± 1.86
**AP(24 h)**	6	5.67 ± 2.42	22.33 ± 5.50	0.217 ± 0.028	27.44 ± 7.41
**AP(48 h)**	6	6.50 ± 1.52	23.67 ± 7.47	0.219 ± 0.028	25.51 ± 1.62

Table S4. ***Group-level descriptive statistics (mean ± SD) for MLEUS, MoLUS,***

histology, and lung wet-to-dry (W/D) ratios across Sham, 6 h, 24 h, and 48 h groups. This table provides the exact summary values requested by the reviewers, allowing clear comparison across imaging, histological, and gravimetric markers. MLEUS increased significantly after aspiration and demonstrated temporal stability. MoLUS showed higher absolute scores but lacked region-specific nuance. Histology and W/D values reflected progressive pathophysiology consistent with prior murine aspiration models.

Supplementary Table S5: Statistical analysis summary for scoring system comparison.

**Analysis**	**Comparison**	**Test Used**	**Statistic**	**p-value**	**Effect Size**	**Summary Interpretation**
**Group differences (4 groups)**	MoLUS total across Sham, 6 h, 24 h, 48 h	One-way ANOVA	F(3,20) = **7.47**	**0.0014**	η^2 = **0.53** (large)	MoLUS detects group-level injury progression but lacks region specificity.
	MLEUS total across Sham, 6 h, 24 h, 48 h	One-way ANOVA	F(3,20) = **12.60**	**7.5×10^{-5}**	η^2 = **0.65** (large)	MLEUS shows stronger group discrimination than MoLUS.
**Paired comparison (same mouse)**	MLEUS vs MoLUS total	Wilcoxon signed-rank test	W = **0**, Z = **–4.29**	**< 0.0001**	Cohen's d = **2.35** (very large)	MoLUS systematically overestimates severity compared with MLEUS.
**Correlation vs Histology**	MLEUS vs Histology	Pearson correlation	r = **0.293**	0.164	—	Weak but directionally consistent correlation.
	MoLUS vs Histology	Pearson correlation	r = **0.034**	0.87	—	No association; MoLUS unsuitable for AP injury grading.
**Correlation vs W/D ratio**	MLEUS (time-point means) vs W/D	Spearman correlation	ρ = **0.20**	0.80	—	Weak association; exploratory only (n=4 timepoints).
	MoLUS (time-point means) vs W/D	Spearman correlation	ρ = **0.04**	0.96	—	No meaningful correlation.
**Inter-method association**	MLEUS vs MoLUS	Pearson correlation	r = **0.724**	< 0.001	—	Systems capture related aeration signals, but

						MoLUS lacks histologic relevance.
**Variance comparison**	MoLUS vs MLEUS variance	Levene's test	F = **18.2**	**0.0003**	—	MoLUS has significantly higher variability, suggesting artifact sensitivity.

Table S5. ***Summary of statistical tests comparing MLEUS and MoLUS, including ANOVA/Kruskal–Wallis tests, paired Wilcoxon analyses, and effect-size estimates (η^2, Cohen's d).*** These analyses confirm that although both scoring systems differentiate aspiration from Sham, only MLEUS demonstrates meaningful association with histological injury. MoLUS exhibited systematic overestimation and poor construct validity. Effect-size analyses further highlight the diagnostic advantages of MLEUS for region-dependent lung injury in small-animal models.

Supplementary Figure S1: Paired comparison between MLEUS and MoLUS scores.

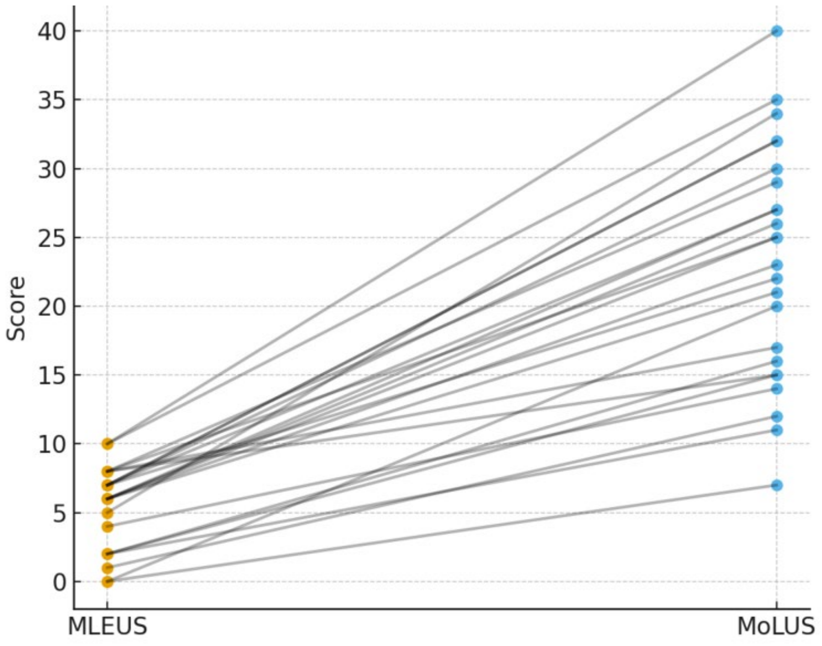

Figure S1. ***Paired comparison between the Modified Lung Edema Ultrasound Score (MLEUS) and the Mouse Lung Ultrasound Score (MoLUS) in all mice (n = 24).*** Each dot represents an individual mouse, with lines connecting paired MLEUS and MoLUS values. MoLUS consistently produced higher injury scores than MLEUS across all time points, reflecting its tendency to inflate severity due to diffuse B-line sensitivity and lack of region-specific zoning. In contrast, MLEUS demonstrated a narrower distribution that aligned more closely with anatomical heterogeneity in aspiration pneumonia. This comparison highlights systematic differences between the two scoring systems and underscores the importance of anatomical resolution in small-animal LUS.

Supplementary Figure S2: Bland–Altman analysis comparing MoLUS and MLEUS.

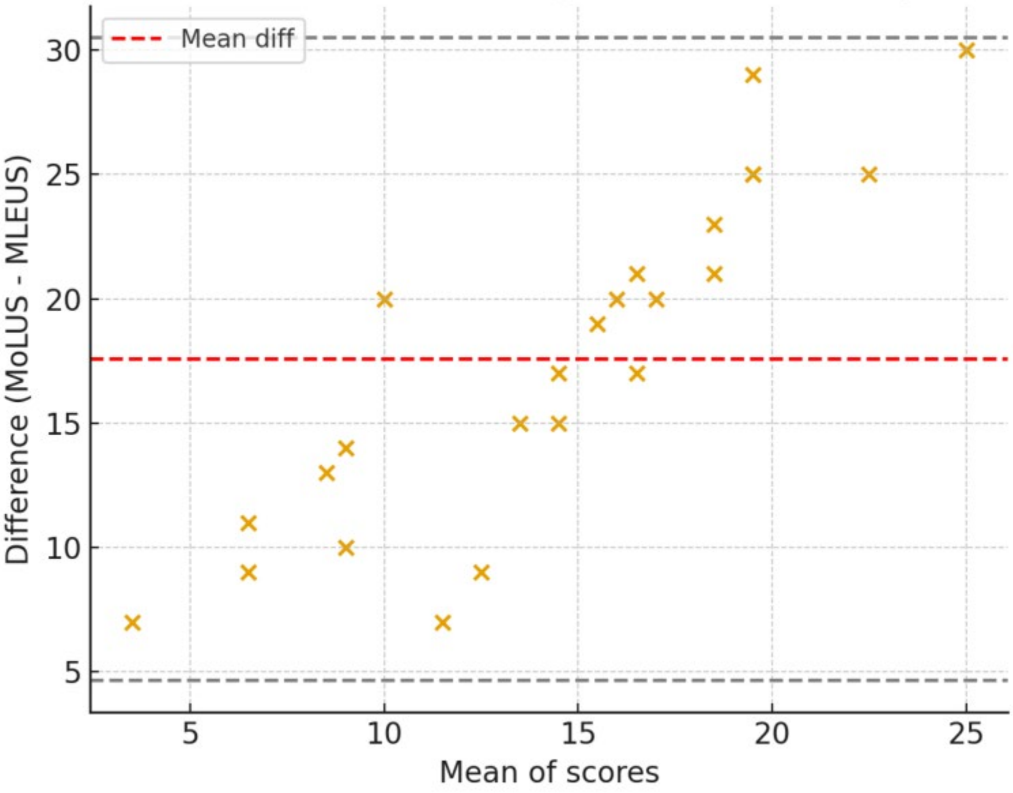

Figure S2. ***Bland–Altman plot depicting agreement between MoLUS and MLEUS scoring systems.*** The mean difference (MoLUS – MLEUS) is indicated by the red dashed line, with 95% limits of agreement shown in gray. MoLUS

overscored MLEUS by an average of approximately 17 points, with wide limits of agreement, indicating that the two scoring systems are not interchangeable. Systematic overestimation by MoLUS arises from its non-zonal design and susceptibility to diffuse artifacts, whereas MLEUS provides region-weighted information tailored to the gravity-dependent, heterogeneous injury pattern characteristic of aspiration pneumonia.

Supplementary Figure S3: Correlation heatmap among MLEUS, MoLUS, and histology.

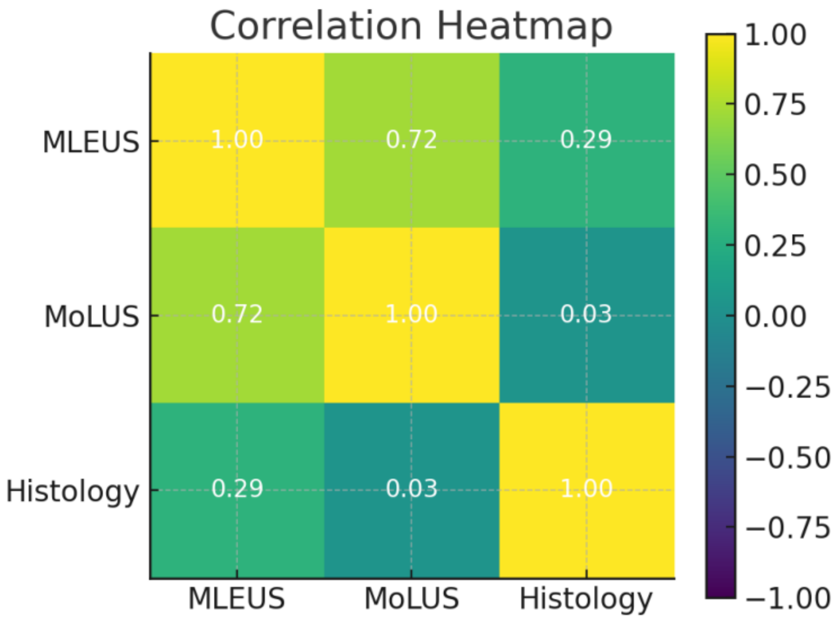

Figure S3. ***Correlation heatmap comparing MLEUS, MoLUS, and histology injury scores.*** Pearson correlation coefficients are displayed numerically within each cell. MLEUS demonstrated a moderate positive correlation with histological severity (r = 0.29), whereas MoLUS showed almost no association (r = 0.03). Both systems exhibited high inter-correlation (r = 0.72), indicating shared sensitivity to ultrasound artifacts, but only MLEUS preserved biologically meaningful alignment with tissue-level injury. These findings further support the superior anatomical relevance and diagnostic value of the MLEUS framework.
